# Clinical, Radiographic, and Inflammatory Peri-Implant Parameters around Narrow Diameter Implant Crowns among Prediabetic and Non-Diabetic Subjects

**DOI:** 10.3390/medicina58121839

**Published:** 2022-12-14

**Authors:** Abdulaziz Alsahhaf, Yasser Alali, Sultan Albeshri, Abdulaziz K. A. Subayt, Abdullah Alomayri, Tariq Abduljabbar, Fahim Vohra

**Affiliations:** 1 Department of Prosthetic Dental Science, College of Dentistry, King Saud University, Riyadh 11545, Saudi Arabia; 2 Department of Maxillofacial Surgery, College of Dentistry, King Saud University, Riyadh 11545, Saudi Arabia; 3 Department of Periodontics and Community Dentistry, College of Dentistry, King Saud University, Riyadh 11545, Saudi Arabia; 4 College of Dentistry, King Saud University, Riyadh 11545, Saudi Arabia

**Keywords:** prediabetes, narrow-diameter dental implants, interleukins, peri-implant crevicular fluid, splinted crowns

## Abstract

*Background and Objectives*: To compare the clinical, radiographic, and inflammatory peri-implant parameters around narrow diameter implants (NDI) supported single and splinted crowns in non-diabetics and prediabetes. *Materials and Methods*: The clinical and radiographic parameters and the levels of IL-6 and TNF-α in the peri-implant crevicular fluid (PICF) of narrow diameter single (NDISCs) and splinted (NDISPs) crown implants were assessed both in non-diabetics and participants with prediabetes. The glycemic state of the patient was assessed using glycated hemoglobin (HbA1c) levels. The peri-implant soft tissue indices (Plaque index (PI), bleeding on probing (BoP), probing depth (PD)) and marginal bone loss were recorded and compared between the groups. Success of the prosthesis was assessed by the frequency of technical complications and patient satisfaction. Inter-group comparison was performed using ANOVA (one-way analysis of variance) while the normal distribution of dependent variables was calculated using Shapiro–Wilk. A *p*-value of less than 0.05 was considered to be statistically significant. *Results*: Sixty participants (30 non-diabetics and 30 with prediabetes) with a total of 178 (118 NDISCs and 60 NDISPs) platform-switched NDIs were a part of the study. Of the 118 NDISCs, 56 were placed in the non-diabetic individuals and 62 were placed in the prediabetes group whereas 30 NDISPs each were placed in both the study groups. The clinical parameters of PI, BoP and PD in the single crown and splinted crown groups showed comparable results. However, a statistically significant difference (*p*-value of less than 0.05) in PI, BoP and PD and in the values of IL-6 and TNF-α was found when a comparison was made between the non-diabetes and prediabetes group. A total of 91% of the patients were satisfied with the esthetics of the implants while 79% of the patients showed satisfaction with function. *Conclusions*: All the clinical and radiographic parameters were statistically similar in both single and splinted types of narrow diameter implants. However, the bone loss, probing depth, plaque index, and levels of inflammatory markers were statistically higher in prediabetes as compared to non-diabetes implying that a slight hyperglycemic state impacts peri-implant health.

## 1. Introduction

According to the International Diabetes Federation (IDF), 463 million people were inflicted with diabetes mellitus in 2020 [1]. According to the World Health Organization (WHO) reports, 220 million people, or 2.8% of the total world population is debilitated by the global pandemic of diabetes. Unfortunately, the numbers are expected to double by 2030, keeping in view the rate at which the incidence is increasing [2]. The disrupted metabolism in diabetes causes micro and macrovascular complications adversely affecting the quality of life. Oral diseases progress faster in diabetic individuals leading to a vast array of complications [3]. 

It is a well-established fact that chronic hyperglycemia associated with diabetes accelerates periodontal inflammation and worsens overall periodontal health [4,5,6]. Poor periodontal status in proportion to poor glycemic control is a result of hyperglycemia-induced accelerated expression of inflammatory mediators such as Interleuikin-6 (IL-6), Tumor necrosis factor-Alpha (TNF-α), and toll-like receptors. Additionally, the advanced glycation end products (AGEs) produced as a result of persistent hyperglycemia accumulate in the periodontal tissues, causing pro-inflammatory and pro-oxidative cellular changes to augment the periodontal tissue destruction [6]. 

Prediabetes is an intermediate hyperglycemic state whereby glycemic parameters are above the normal threshold and below the diabetic cut-off values [7]. The WHO labels prediabetes using two diagnostic tests, namely the fasting plasma glucose (FPG) and the oral glucose tolerance test (OGTT). FPG values between 110 and 125 mg/dL and impaired glucose tolerance (2 h plasma glucose of 140–200 mg/dL after intake of 75 g of oral glucose load) categorize prediabetes. The American Diabetes Association (ADA), however, requires an additional hemoglobin A1c value between 5.7% and 6.4% to define prediabetes [8]. Nevertheless, the lack of uniformity in the diagnostic criteria does not make the condition any less trivial owing to two major reasons. Firstly, as many as 10% of people with prediabetes, annually, are at risk of becoming inflicted with diabetes, and secondly, there is a high burden of cardiometabolic risk factors and risk of microvascular complications such as retinopathy, nephropathy, and neuropathy in prediabetes [9]. 

Implant dentistry has been substantially researched and implant-supported restorations are now the oft-used treatment of choice for tooth loss where the surrounding hard and soft tissues are healthy and oral hygiene is adequately maintained. Conventional implants have been widely used as a predictable treatment option for the restoration of masticatory function and aesthetics in edentulous patients [10]. However, the treatment outcomes have been ineffective in cases where the mesiodistal bone thickness or lingual-occlusal height of the bone is inadequate [11]. This problem has been recently tackled by the introduction of narrow diameter implants (NDI) which eludes complex surgical bone augmentation procedures [12]. Implant-supported single crown restorations (NDISC), as the name implies, are prostheses with a single crown supported by an implant and are popularly used for their advantages of better hygiene control, greater patient comfort and ease of repair. Splinted crowns (NDISP) are implant-supported restorations with splinted crowns to ensure a better stress distribution, and this is a relatively newer concept. 

Although dental implants were previously not recommended in type 2 diabetes owing to the associated microvascular complexities, it has now been proven that dental implants display optimal stability and osseointegration under stringent glycemic control [13]. However, the peri-implant tissues are susceptible to endogenous oral infections, analogous to periodontitis of natural teeth [14]. Peri-implant disease has been identified and quantified in previous research studies, using marginal bone loss (MBL), probing depth (PD), and bleeding on probing (BOP) [15]. 

Although research has been completed to compare the NDI with the regular diameter implant and NDISCs with the NDISPS at various jaw positions [16,17], there is no research comparing the two varieties of implants in prediabetes and diabetes. Considering the high conversion of prediabetes into diabetes and the increasing number of people in need of dental implants, this cross-sectional study aimed to study and compare the clinical and radiographic parameters around NDISCs and NDISPs in prediabetes and normoglycemic individuals. Another aim of the current study was to study and compare interleukin-6 (IL-6) and tumor necrosis factor-alpha (TNF-α) in the peri-implant crevicular fluid around NDISCs and NDISPs in non-diabetics and prediabetics. The null hypothesis states that there will be no difference in the clinical and radiographic parameters and values of IL-6 and TNF-α around NDISCs and NDISPs in nondiabetics and prediabetes. 

## 2. Materials and Methods

### 2.1. Study Design and Ethical Consideration

This was a cross-sectional study design carried out according to the principles laid in the Declaration of Helsinki (2013). The study protocol was reviewed and approved by the research ethics committee of specialist dental practice and research center (UDCRC-RB-022-21) April 2021. The clinical and radiographic peri-implant parameters were analyzed along with the cytokine analysis of the peri-implant crevicular fluid. Before giving their informed consent, the participants filled out a detailed form containing the purpose and methodology of the study and requiring details of the biodata, brushing habits and medical well-being of the participants.

### 2.2. Inclusion and Exclusion Criteria

Prediabetics (HbA1c between 5.7 and 6.4) and non-diabetics (HbA1c less than 5.7) were both included in the study if they were aged 25 years and above, had NDISPs or NDISCs and had a follow-up of 5 years and more. Bone quality of category D3 (thin cortical and dense cancellous) was considered for implant placement. Participants were excluded if they had gone through bone-augmentation surgical procedures, had compromised periodontal health, were chronic smokers, were completely edentulous or did not have comparative radiographic data of baseline.

### 2.3. Screening of Implants and Prosthesis

Implants with moderately rough surfaces and a narrow diameter of 2.9 mm were placed at bone level. A detailed radiographic and clinical assessment was performed by one trained examiner who noted down the age and gender of patients, number of implants, type of restorations (screw-retained/cement-retained), and location and length of implant and peri-implant conditions.

### 2.4. Clinical Peri-Implant Parameters

With the inter examiner reliability of 0.93 (kappa), a trained examiner assessed the dental implants and recorded the baseline data. All six sites (mesiobuccal, distolingual, mid-lingual, distobuccal, mid-buccal, mesiolingual) were examined to check the plaque index (PI) and bleeding on probing (BoP). The scoring was completed as 1 if plaque or BoP was present and 0 if absent. To record the probing depth to the nearest mm, a graded periodontal probe (UNC-15 Hu-Friedy, Chicago, IL, USA) was used [18].

### 2.5. Radiographic Assessment

Using the blinding method, a trained examiner performed the radiographic assessment. Periapical radiographs were taken digitally and evaluated on a display screen (Samsung SyncMaster digital TV monitor, Seoul, Republic of Korea) using an image analyzer (Scion Image Analyzer, Scion, Frederick, MD, USA). The total vertical distance from the crest of the alveolar bone to the highest point on the supracrestal part of the dental implant was measured to calculate the peri-implant bone loss [19].

### 2.6. Analysis of Peri-Implant Crevicular Fluid for Inflammatory Markers

The supragingival plaque over the implants was cleaned with gauze. Cotton rolls were used to isolate the area around the implants. The PICF was then collected using paper strips (Periopaper^®^, Oraflow, Hewlett, NY, USA) which were aseptically placed one millimeter in the peri-implant sulcus for 30 s [20]. Contaminated samples were discarded. After collection, the fluid volume was measured with a Periotron^®^, and the paper strips were inserted into tubes containing 300 μL of PBS buffer and protease inhibitor (100 mL/1 mL of fluid Sigma-Aldrich). After 30 min, the strips were discarded, and the solution was centrifuged at 3000× *g* for 5 min and frozen at −70 °C pending analysis. Following the collection of PICF, a high-sensitivity multiplex map human cytokine immunoassay (Millipore Corporation^®^, Billerica, MA, USA) was used to measure the levels of IL-6 and TNF-α. 

### 2.7. Patient Satisfaction

A questionnaire containing questions related to the function and esthetics of restoration was filled out by all participants. The participants were required to answer sections containing the Likert scale ranging from ‘extremely satisfied’ to ‘extremely dissatisfied’.

### 2.8. Assessment of Technical Complications

A detailed examination was carried out to check for the loosening or fracture of the implant or an abutment screw, a chipping of a crown, and/or loss of retention.

### 2.9. Statistical Analysis

Power analysis was conducted based on the alpha level set at 0.05, with a power of 80%, and medium effect size of 0.2 for both groups, and compensating for a 15% attrition rate of the study participants, a total of 30 participants per group were included [21]. The statistical analysis was carried out using specialized software (SPSS v 21, Chicago, IL, USA). A *p*-value of less than 0.05 was considered significant. Means and standard deviations were generated. Demographic parameters such as mean age, gender, mean HbA1c, and a family history of diabetes were computed using Mann–Whitney, while *p* values for oral hygiene practice including toothbrushing frequency were generated using the Chi-square test. Inter-group comparison of clinical, radiographic, and inflammatory parameters was performed using ANOVA (one-way analysis of variance) while the normal distribution of dependent variables was calculated using Shapiro–Wilk.

## 3. Results

Table 1 depicts the demographics of the participants along with their family history of diabetes and brushing habits. A total of 60 patients participated in the study out of which 30 were non-diabetic while 30 subjects had prediabetes. In the non-diabetic group, 20 were males and 10 were females, whereas in the prediabetes group, there were 18 males and 12 females. The mean HbA1C in the non-diabetic group was 4.5 and that in the prediabetes group was 6.8. Nine individuals in the non-diabetic group had a family history of diabetes whereas 21 individuals reported some form of diabetes in the family. The majority of the participants in both groups brushed their teeth once daily. A significant difference was noted between mean HbA1c values (*p* = 0.0376), family history of diabetes (*p* = 0.001) and toothbrushing practices (*p* = 0.012) between non-diabetic and prediabetic groups (Table 1).

Table 2 shows the characteristics of the implants that were used for the study. A total of 178 (118 NDISCs and 60 NDISPs) platform-switched NDIs with a moderately rough surface were evaluated. Of the 118 NDISCs, 56 were placed in the non-diabetic individuals and 62 were placed in the prediabetes group whereas 30 NDISPs each were placed in both the study groups. All the implants used had a narrow diameter of 2.9 mm, were placed at bone level, and loaded at approximately 3 months. Out of the 178 implants used, 124 were 10 mm in length and 57 were 12 mm in length. All the crowns were screw-retained. In the non-diabetic group, NDISCs were in function for 12.4 years while NDISPs were in function for 13.9 years. In the prediabetes group, NDISCs were in function for 14.1 years whereas NDISPs were functional for 13.5 years. 

Table 3 demonstrates the soft and hard tissue parameters including PI, BoP, PD, and CBL around the NDIs in the non-diabetic and prediabetes group. PI and BoP in the non-diabetic group were measured as 25.4% and 12.3%, and 28.3% and 15.6% around NDISCs and NDISPs, respectively. In the prediabetes group, the PI and BoP was recorded as 36.7% and 26.8% and 39.5% and 28.2% around NDISCs and NDISPs, respectively. The mean PD in the non-diabetic group was 3.3 mm and 3.1 mm around NDISCs and NDISPs, respectively. The mean PD in the prediabetes group was 3.9 mm around NDISCs and 3.8 mm around NDISPs. The mesial crestal bone level in the non-diabetic group was 1.13 mm and 1.05 mm around NDISCs and NDISPs, respectively. In the T2DM group, the bone level was 1.67 mm and 1.62 mm around NDISCs and NDISPs, respectively. The distal crestal bone level in the non-diabetic group was recorded as 1.19 mm and 1.11 mm around NDISCs and NDISPs, respectively. In the prediabetes group the distal crestal bone was measured as 1.59 mm and 1.71 mm around NDISCs and NDISPs, respectively. The clinical readings did not show any significant difference when an inter-group comparison between NDISC and NDISP was made. However, there was a significant difference in all parameters between the non-diabetic and prediabetes groups.

Table 4 shows the levels of the biomarkers IL-6 and TNF-α in pg/mL in the blood samples of participants of both groups. In the non-diabetics, the value of IL-6 in NDISC was 85 pg/mL and that of TNF-α was 47 pg/mL while the value of IL-6 and TNF-α in NDISP was 94 pg/mL and 63 pg/mL, respectively. The levels of IL-6 and TNF-α in the single crown group of prediabetes were 138 pg/mL and 98 pg/mL, respectively. In the splinted crown subgroup of prediabetes, the value of IL-6 was 154 pg/mL and that of TNF-α was 115 pg/mL. There was no statistical difference in the single and splinted crown groups in both non-diabetes and prediabetes. However, when an inter-group comparison was made between non-diabetes and diabetes, the values of both IL-6 and TNF-α were significantly different among both the NDISCs and NDISPs.

Table 5 depicts the peri-implant bone loss and technical complications in both single and splinted crowns in both participants having prediabetes and participants with no diabetes. The most common complication reported was chipping and loosening of crowns. A total of 28.6% of the participants without diabetes and 15.8% of the prediabetes participants complained of technical issues in single crown restoration. Among the non-diabetics, 21.4% and among the prediabetes, 34.9% of the participants reported complications associated with splinted crowns. There was a significant difference in the technical complications associated with single and splinted crowns in both the non-diabetes and prediabetes group. However, the difference in the peri-implant bone loss was statistically non-significant in the two groups.

Table 6 depicts the overall patient satisfaction. A total of 91% of the patients were satisfied with the esthetics of the implants while 79% of the patients showed satisfaction with function. A total of 9% of the patients were not satisfied with the esthetics whereas 21% of the patients showed dissatisfaction with the function of the implants.

## 4. Discussion

To the best of the authors’ knowledge of the indexed literature, this is a pioneering study that evaluated and compared the clinical and radiographic parameters along with the crevicular fluid titers of IL-6 and TNF-α in implants placed in normoglycemic individuals and compared the same with participants having prediabetes. A considerable percentage of participants showed satisfaction and had few technical complications, demonstrating overall success.

The prediabetes status of the study participants was confirmed with the help of the patients’ medical records of HBA1C measurement. We expected to see worsened clinical and radiographic picture and elevated levels of IL-6 and TNF-α in the PICF of prediabetes as compared to the non-diabetic individuals. As hypothesized, the PI, BoP, PD, and the crestal bone loss were significantly different in the prediabetes group as compared to the normoglycemic individuals. This is in accordance with previous studies by Al Amri et al., Mokeem et al. and others [19,22,23] and can be explained by the observation that long-standing hyperglycemia leads to increased numbers of AGEs in the periodontal tissues causing inflammation and eventually bone loss [23,24]. Although the glycosylated hemoglobin levels in prediabetes are lower than that of diabetics, it is much more than that in non-diabetics. The relatively high HbA1C titers in prediabetes delay the maturation and maintenance of the extracellular matrix in the tissues surrounding the implants, contributing to more tissue destruction [25]. For similar reasons, the TNF-α and IL-6 levels were significantly higher in the PICF of the prediabetes group. The AGEs interact with their respective receptors to trigger the expression of destructive inflammatory mediators such as IL-6 and TNF-α in the tissues crevicular fluid and resultantly, activate a cascade of inflammatory reactions. This leads to worsening inflammation and loss of bone surrounding dental implants [26]. Recent systematic reviews have proven that if blood sugar levels are maintained, osteogenesis in the peri-implant tissues can be warranted [27,28] which in turn could help maintain the clinical and radiographic parameters.

Although Kaplan–Meier survival analysis could have given precise numbers on the implant survival rates, the 5 year follow up showed considerably sound peri-implant conditions [29]. This could be explained by the fact that all subjects maintained stringent oral hygiene and followed a 6-month dental prophylaxis program. This program ensured regular visits to cleanse plaque from teeth and implant surfaces and to confirm if the participants were regularly following oral hygiene maintenance measures. Interestingly, there was a significant difference in the oral hygiene practices between the two groups which may have impacted our results. Salman et al., proved that periodontal parameters were strikingly different between diabetic and non-diabetic even when the oral hygiene measures were similar [30]. According to another study, the detrimental effect of hyperglycemia on periodontal tissues could be controlled by following strict oral hygiene measures [31]. Studies have proven that a 100% success rate is achievable even in hyperglycemic conditions, provided the oral hygiene is meticulously maintained [32,33,34]. However, the oral hygiene maintenance could be standardized in future studies to help draw definite conclusions.

The null hypothesis was partially rejected. Although there was a difference in the radiographic and clinical readings between the normoglycemic and heteroglycemic groups, the differences between the NDISCs and the NDISPs were non-significant. This was in keeping with a previous study that concluded that there was clinically un-meaningful difference in bone loss between the two NDI groups [35]. This possibly indicates that both NDISCs and NDISPs can be alternately used without expecting peri-implant bone loss. 

This is the first study of its kind which has evaluated and compared the prognostic parameters around narrow diameter implants in subjects with prediabetes and non-diabetic subjects. The significant difference in the clinical and laboratory parameters between the groups indicates that the hyperglycemic state in prediabetes could be potential cause of periimplantitis. Therefore, it is imperative that necessary measures should be taken to control chronic the hyperglycemic state and to optimize oral health that could ensure the success and longevity of the dental implants. Nevertheless, there were certain limitations in the current study, which can be considered in similar studies in the future. It was a cross-sectional study and the HbA1c was noted at one point in time, overlooking the fluctuations that could have taken place during the 5-year follow up. It is therefore advisable that patients having HBA1c above the normal threshold such as those with prediabetes and diabetes and having dental implant therapy should have their glycemic levels repeatedly monitored and accordingly maintain it within the acceptable range. The accurate performance of implants is determined through success and survival rates which were not explored in this study. Strict inclusion criteria were employed for the study such as absence of smoking habits, no bone augmentation procedure and sound periodontal health. These factors could possibly have impacted the study outcome and results could be different in cases where these criteria are not met. Future prospective studies could warrant confirmatory outcomes. 

## 5. Conclusions

All the clinical and radiographic parameters were statistically similar in both single and splinted types of narrow diameter implants. However, the bone loss, probing depth, plaque index and levels of IL-6 and TNF-α in the PICF were statistically higher in prediabetes as compared to non-diabetes, implying that hyperglycemia induces irreversible peri-implant bone alterations. 

## Figures and Tables

**Table 1:** Patient demographics.

	Non-Diabetic	Prediabetes	*p*-Value
Number of patients	30	30	1.000 *
Mean Age in years	52.5 ± 4.4	53.5 ± 4.5	0.712 *
Male/Female	20/10	18/12	0.844 *
Mean HbA1c (SD)	4.5 ± 0.9	6.8 ± 1.8	0.0376 *
Family history of diabetes (*n*)	9	21	0.001 *
Tooth brushing (%)			
Once daily	69	88	
Twice daily	31	12	0.012 **

* *p*-values generated using Mann-Whitney test. ** *p*-values generated using Chi-Square test.

**Table 2:** Implant-related description.

Parameters	Non-Diabetic		Prediabetes	
	NDISC	NDISP	NDISC	NDISP
Total number of implants	56	30	62	30
Depth of placement	BL	BL	BL	BL
Implant design	PS with moderately rough surfaces	PS with moderately rough surfaces	PS with moderately rough surfaces	PS with moderately rough surfaces
Implant length (10/12 mm) and diameter	40/16	18/12	46/16	20/10
Implant loading after placement (in months)	3.3 ± 0.6	3.2 ± 0.5	3.2 ± 0.4	3.4 ± 0.3
Duration of implants in function (in years)	12.4 ± 2.0	13.9 ± 1.8	14.1 ± 3.1	13.5 ± 2.7

NDISC: narrow-diameter implant supporting single crowns, NDISP: narrow-diameter implant supporting splinted crowns, BL: bone-level, SR: screw-retained, CR: cement-retained.

**Table 3:** Peri-implant soft tissue status among non-diabetic and prediabetic groups and restorations.

Peri-Implant Parameters	Non-Diabetic		Prediabetes	
	NDISC	NDISP	NDISC	NDISP
Plaque index (% of sites)	21.7 ± 6.3 ^A^	29.6 ± 8.4 ^A^	35.9 ± 9.5 ^B^	41.2 ± 11.2 ^B^
Bleeding on probing (% of sites)	18.4 ± 6.8 ^A^	21.8 ± 9.9 ^A^	24.4 ± 10.6 ^B^	35.3 ± 16.5 ^C^
Probing depth (in mm)	3.1 ± 0.7 ^A^	3.3 ± 0.8 ^A^	4.0 ± 1.2 ^B^	4.2 ± 1.6 ^B^
Crestal bone levels (in mm)				
Mesial	0.8 ± 0.3 ^A^	1.0 ± 0.5 ^A^	1.6 ± 0.8 ^B^	1.7 ± 1.0 ^B^
Distal	0.9 ± 0.4 ^A^	1.1 ± 0.4 ^A^	1.7 ± 0.9 ^B^	1.8 ± 0.9 ^B^

Dissimilar upper-case letters indicate statistical significance at *p* < 0.05.

**Table 4:** Levels of IL-6 and TNF-α from the study groups.

Biomarkers	Non-Diabetic		Prediabetes	
	NDISC	NDISP	NDISC	NDISP
IL-6 (pg/mL)	85 ± 42 ^A^	94 ± 58 ^A^	138 ± 79 ^B^	154 ± 84 ^B^
TNF-α (pg/mL)	47 ± 26 ^A^	63 ± 29 ^A^	98 ± 40 ^B^	115 ± 47 ^B^

Different superscript alphabets denote statistical significance.

**Table 5:** Influence of type of prostheses on technical complications and peri-implant bone loss.

	Technical Complications	Peri-Implant Bone Loss
Non-diabetic		
Single crown	28.6%	0.8 ± 0.3
Splinted crown	21.4%	0.9 ± 0.2
*p*-values	0.066	0.164
Prediabetes		
Single crown	15.8%	1.5 ± 0.5
Splinted crown	34.9%	1.6 ± 0.7
*p*-values	0.0018	0.429

**Table 6:** Overall patient satisfaction.

	Satisfied Patients (%)	Unsatisfied Patients (%)
Esthetics	91%	9%
Function	79%	21%
Overall satisfaction (mean VAS and SD)	18.3 ± 6.7	

## Data Availability

The data are available on contact from the corresponding author.

## References

[1] International Diabetes Federation—Home (idf.org). https://idf.org/.

[2] World Health Organization. https://www.who.int/health-topics/diabetes/diabetes#tab=tab_1.

[3] Alhazmi Y.A., Parveen S., Alfaifi W.H., Najmi N.M., Namazi S.A., Abuzawah L.H., Mashhour N.M. (2022). Assessment of Knowledge, Attitude and Practice of Diabetic Patients Towards Oral Health: A Cross-sectional Study. World J. Dent..

[4] Graves D.T., Ding Z., Yang Y. (2020). The impact of diabetes on periodontal diseases. Periodontology.

[5] Alasqah M., Mokeem S., Alrahlah A., Al-Hamoudi N., Abduljabbar T., Akram Z., Vohra F., Javed F. (2018). Periodontal parameters in prediabetes, type 2 diabetes mellitus, and non-diabetic patients. Braz. Oral. Res..

[6] Llambés F., Arias-Herrera S., Caffesse R. (2015). Relationship between diabetes and periodontal infection. World J. Diabetes.

[7] Hostalek U. (2019). Global epidemiology of prediabetes-present and future perspectives. Clin. Diabetes Endocrinol..

[8] Echouffo-Tcheugui J.B., Selvin E. (2021). Pre-diabetes and what it means: The epidemiological evidence. Annu. Rev. Public Health.

[9] Bansal N. (2015). Prediabetes diagnosis and treatment: A review. World J. Diabetes.

[10] Lemos C.A.A., Verri F.R., Santiago Junior J.F., de Souza Batista V.E., Kemmoku D.T., Noritomi P.Y., Pellizzer E.P. (2018). Splinted and nonsplinted crowns with different implant lengths in the posterior maxilla by three-dimensional finite element analysis. J. Healthc. Eng..

[11] Menini M., Pesce P., Delucchi F., Ambrogio G., Canepa C., Carossa M., Pera F. (2022). One-stage versus two-stage technique using two splinted extra-short implants: A multicentric split-mouth study with a one-year follow-up. Clin. Implant Dent. Relat. Res..

[12] Ma M., Qi M., Zhang D., Liu H. (2019). The clinical performance of narrow diameter implants versus regular diameter implants: A meta-analysis. J. Oral Implantol..

[13] Javed F., Romanos G.E. (2009). Impact of diabetes mellitus and glycemic control on the osseointegration of dental implants: A systematic literature review. J. Periodontol..

[14] Belibasakis G.N., Manoil D. (2021). Microbial community-driven etiopathogenesis of peri-implantitis. J. Dent. Res..

[15] Jiang X., Zhu Y., Liu Z., Tian Z., Zhu S. (2021). Association between diabetes and dental implant complications: A systematic review and meta-analysis. Acta Odontal. Scand..

[16] Alrabiah M., Al Deeb M., Alsahhaf A., AlFawaz Y.F., Al-Aali K.A., Vohra F., Abduljabbar T. (2020). Clinical and radiographic assessment of narrow-diameter and regular-diameter implants in the anterior and posterior jaw: 2 to 6 years of follow-up. J. Periodont. Implant Sci..

[17] Assaf A., Saad M., Hijawi S. (2022). Use of narrow-diameter implants in the posterior segments of the jaws: A retrospective observational study of 2 to 11 years. J. Prosthet. Dent..

[18] Avetisyan A., Markaryan M., Rokaya D., Tovani-Palone M.R., Zafar M.S., Khurshid Z., Vardanyan A., Heboyan A. (2021). Characteristics of periodontal tissues in prosthetic treatment with fixed dental prostheses. Molecules.

[19] Al Amri M.D., Abduljabbar T.S., Al-Kheraif A.A., Romanos G.E., Javed F. (2017). Comparison of clinical and radiographic status around dental implants placed in patients with and without prediabetes: 1-year follow-up outcomes. Clin. Oral Implant. Res..

[20] Heboyan A., Syed A.U.Y., Rokaya D., Cooper P.R., Manrikyan M., Markaryan M. (2020). Cytomorphometric analysis of inflammation dynamics in the periodontium following the use of fixed dental prostheses. Molecules.

[21] Messias A., Rocha S., Wagner W., Wiltfang J., Moergel M., Behrens E., Nicolau P., Guerra F. (2019). Peri-implant marginal bone loss reduction with platform-switching components: 5-Year post-loading results of an equivalence randomized clinical trial. J. Clin. Periodontol..

[22] Mokeem S., Alfadda S.A., Al-Shibani N., Alrabiah M., Al-Hamdan R.S., Vohra F., Abduljabbar T. (2019). Clinical and radiographic peri-implant variables around short dental implants in type 2 diabetic, prediabetic, and non-diabetic patients. Clin. Implant Dent. Relat. Res..

[23] Al-Sowygh Z.H., Ghani S.M.A., Sergis K., Vohra F., Akram Z. (2018). Peri-implant conditions and levels of advanced glycation end products among patients with different glycemic control. Clin. Implant Dent. Relat. Res..

[24] Akram Z., Alqahtani F., Alqahtani M., Al-Kheraif A.A., Javed F. (2020). Levels of advanced glycation end products in gingival crevicular fluid of chronic periodontitis patients with and without type-2 diabetes mellitus. J. Periodontol..

[25] Chambrone L., Palma L.F. (2019). Current status of dental implants survival and peri-implant bone loss in patients with uncontrolled type-2 diabetes mellitus. Curr. Opin. Endocrinol. Diabetes Obes..

[26] Celik D., Kantarci A. (2021). Vascular changes and hypoxia in periodontal disease as a link to systemic complications. Pathogens.

[27] Patel V., Sadiq M.S., Najeeb S., Khurshid Z., Zafar M.S., Heboyan A. (2022). Effects of metformin on the bioactivity and osseointegration of dental implants: A systematic review. J. Taibah Univ. Med. Sci..

[28] Akram Z., Vohra F., Javed F. (2018). Locally delivered metformin as adjunct to scaling and root planing in the treatment of periodontal defects: A systematic review and meta-analysis. J. Periodontal Res..

[29] Chuang S.K., Tian L., Wei L.J., Dodson T.B. (2001). Kaplan-Meier analysis of dental implant survival: A strategy for estimating survival with clustered observations. J. Dent. Res..

[30] Salman B.N., Shabestari S.B., Jam M.S., Tari S.A., Shirinbak I. (2020). Periodontal parameters and oral hygiene in diabetic and nondiabetic adolescents in Zanjan. Med. J. Islam. Repub. Iran.

[31] Haghdoost A., Bakhshandeh S., Ghorbani Z., Namdari M. (2022). The Relationship between Oral and Dental Health Self-care and Hemoglobin A1c in Adults with Diabetes. J. Dent..

[32] Degidi M., Nardi D., Piattelli A. (2012). 10-year follow-up of immediately loaded implants with TiUnite porous anodized surface. Clin. Implant Dent. Relat. Res..

[33] Lin I.C., Gonzalez A.M., Chang H.J., Kao S.Y., Chen T.W. (2011). A 5-year follow-up of 80 implants in 44 patients placed immediately after the lateral trap-door window procedure to accomplish maxillary sinus elevation without bone grafting. Int. J. Oral Maxillofac. Implant..

[34] Baskaran P., Prakash P.S.G., Appukuttan D., Mugri M.H., Sayed M., Subramanian S., Al Wadei M.H.D., Ahmed Z.H., Dewan H., Porwal A. (2022). Clinical and Radiological Outcomes for Guided Implant Placement in Sites Preserved with Bioactive Glass Bone Graft after Tooth Extraction: A Controlled Clinical Trial. Biomimetics.

[35] Vigolo P., Mutinelli S., Zaccaria M., Stellini E. (2015). Clinical evaluation of marginal bone level change around multiple adjacent implants restored with splinted and nonsplinted restorations: A 10-year randomized controlled trial. Int. J. Oral Maxillofac. Implant..

